# A Case of Inflammation of the Labia Pudenda

**Published:** 1861-12

**Authors:** J. B. Hoag


### A CASE OF INFLAMMATION OF THE LABIA PUDENDA.

By J. B. HOAG, M. D.

I have recently treated a case of inflammation of the Labia Pudenda, in an infant aged seven months, which terminated fatally, and which, in my opinion, from the fact that the disease is of rare occurrence and from other considerations, is fraught with more than ordinary interest. During ten years practice in the medical profession, I have never seen but one similar case, which occurred in my own family, during my residence in the city of New York, soon after I commenced practice. My daughter, aged about eighteen or twenty months, was afflicted with inflammation in the private parts, causing much pain and some little constitutional disturbance, which was, by the use of local anti-phlogistic remedies, in a few days removed, and the little sufferer restored to her wonted health.

But, to the case under consideration.

On the 15th of Oct., 1861, I was summoned in haste to the residence of T. Perry, of Tipton county, Ind., some four miles distant from my residence. I found his only child, a girl of seven months of age, giving signs of suffering to no small extent. On enquiry into the history of the case, I learned the following facts: The child had been healthy from the time of its birth, until attacked with the disease from which it was now suffering. The first indications that anything was wrong were, the appearance of slight inflammation about the private parts and indications of extreme pain on emptying the bladder. The father employed W. P. S., an eclectic doctor, who resided nearer him than I did, who made three visits without benefiting the child. He pronounced it inflammation of the kidneys, and though frequently solicited to examine the inflamed parts, refused to do so, assuring the anxious parents and friends that that was of no consequence, and would soon disappear. (I wonder how many cases of Nephritis, in a child of seven months of age, the learned doctor ever saw?) The inflamed parts continuing to swell and the agony of the child increasing under the doctor's treatment, they dismissed him, and I was summoned. I made a careful examination of the region of the kidneys, (which, I was informed, the eclectic doctor had not done,) and could find no evidences of inflammation or tenderness. I discovered but little constitutional disturbance; the tongue was clean, the pulse but little accelerated, the passages natural, and but a light symptomatic fever. The inflamed parts presented a bad appearance, being much swollen, very red and tender. In the hopes that the inflammation could be reduced at once, I directed the mother to keep the parts between the labia clean by frequently washing them with warm water and castile soap; left solutions of Argent Nitras, Plumbi Acetas and Sulph. Zinci, with directions to have a cloth, wet with these solutions, constantly applied to the affected parts. I also directed them to give small doses of Spts. Eth. Nit. occasionally. I did this in order to allay the slight febrile symptoms which were present, and also to act as a diuretic. On my next visit, the next day, I found no indications of the inflammation subsiding, but some little softening in different places. I then directed the parts to be poulticed, and still to be kept clean by frequent ablutions. The next day, I judged that the poultices were hastening suppuration. I intended to return the following day, but did not, in consequence of personal indisposition, and knowing that the child had sufficient medicine to last another day. The next day, which was Saturday, I went rather early and found that the father had gone to Tipton, the county seat, a distance eight miles, for a physician for counsel, and had left particular word for me to remain until he returned. In due time Mr. Perry returned, with my friend, Dr. J. B. White, an excellent young practitioner, a graduate from a medical college in Louisville, Ky. We examined the case together and retired for consultation. He approved of the entire treatment, and recommended small doses of Paregoric occasionally. We also gave the child small doses of carbonate of Iron and Quinine, in order to sustain the system. The child's appetite continued unimpaired, and its strength seemed to be remarkable, considering what it had endured. We agreed to meet there the next day and open the abscess. We did so. Instead of pus, nothing but black, impure blood was discharged. The case was left in my hands. I left the house under the influence of a chill, and was unable to reach home. The next morning I visited my little patient again. I found her much improved. The swelling was much reduced and the appearance of the diseased parts much better. I was hardly able to attend to my duties, and when I left, the father told me that, as I was so much indisposed, he would inform me if the child became worse. Time passed on, and, after a few days, I saw an aunt of the child's, who informed me, in answer to my enquiries respecting it, that "it was getting well as fast as possible." This was indeed good news to me. One week from the day of my last visit, the child's father came for me again. I found the child but little reduced, its strength seeming to keep up remarkably. Each labia had sloughed, leaving a cavity sufficiently large to insert my thumb longitudinally. There was the presence of some pus, but also the appearance of healthy granulations. I continued to give Quinine and Iron, and ordered the parts to be kept clean and poultices applied. I made three consecutive visits, and on each visit the child seemed to be improving. I told I thought it was unnecessary for me to visit it every day, for the most the child needed was careful nursing and good attention. Several days after I heard of the child's death.

What was the cause of its death? Was it from the pus being absorbed and taken up in the circulation? or was it from the strength of the little sufferer failing in spite of the tonics used? or was it from some other cause? Was the treatment correct?

Who will answer the interrogatories?

